# Supplementary material for: High-harmonic generation from artificially stacked 2D crystals
Source: Nanophotonics. 2023 Jan 6;12(2):255–61. doi: 10.1515/nanoph-2022-0595 (PMC11501195; doi:10.1515/nanoph-2022-0595)
Supplement: Supplementary file 1 — Supplementary Material Details [file j_nanoph-2022-0595_suppl.pdf]

Supplementary Information

High-harmonic generation from artificially stacked mm-sized 2D crystals

Christian Heide,[1,2,*] Yuki Kobayashi,[1,2,*] Amalya C. Johnson,[3] Tony F. Heinz,[1,2] David A. Reis,[1,2] Fang Liu,[1,4] and Shambhu Ghimire[1]

[1]*Stanford PULSE Institute, SLAC National Accelerator Laboratory, Menlo Park, CA 94025, USA*

[2]*Department of Applied Physics, Stanford University, Stanford, CA 94305, USA*

[3]*Department of Materials Science and Engineering, Stanford University, Stanford, CA 94305, USA*

[4]*Department of Chemistry, Stanford University, Stanford, CA 94305, USA*

(Dated: November 17, 2022)

* These authors contributed equally.

I. EXPERIMENTAL SETUP.

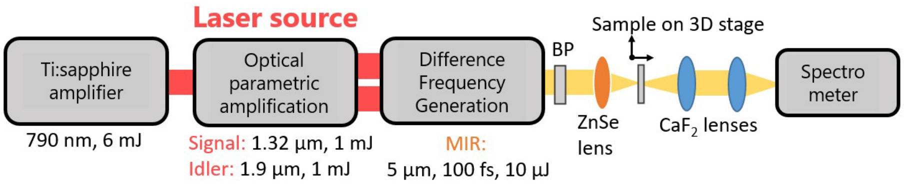

An amplified titanium:sapphire laser system (Evolution, Coherent, Inc., 6 mJ, 45 fs, 790 nm, 1 kHz) is used to pump an optical parametric amplifier (OPA, TOPAS-HE, Light Conversion, Inc.). The signal ($\sim$ 1320 nm) and idler ($\sim$ 1900 nm) from the OPA are mixed in a GaSe crystal (Eksma Optics Inc., z-cut, 0.5 mm thick) for difference-frequency generation. The resulting mid-infrared radiation is cleaned by a bandpass filter, centered at 5.0 μm (Thorlabs Inc., FB5000-500). The polarization state of the MIR beam is controlled with a zero-order MgF_2 half-waveplate. The beam is focused using a ZnSe lens with a focal length of 100 mm. The spot size is $\sim$ 90 μm ($1/e^2$ intensity radius), characterized with a MIR beam profiler (Dataray, WinCamD-IR-BB). The pulse duration has been optimized via HH efficiency by adding BaF_2 and germanium plates and is about 100 fs. The generated high harmonics are collected and focused by CaF_2 lenses and directed into a spectrometer equipped with a thermoelectrically cooled silicon CCD camera (Pixis 400B, Princeton Instruments, Inc.). All HH spectra were measured in a transmission geometry and under ambient conditions. The sample orientation for the linearly polarized MIR laser field was aligned to maximize even-order harmonics.

For the second harmonic generation (SHG) measurements, the signal ($\sim$ 1320 nm) of the OPA is focused to the sample. SHG and fundamental are separated using bandpass filter centered at 660 nm (Thorlabs FB660-10), before sending the beam to the spectrometer.

II. GENERATION AND PROPAGATION OF HIGH-ORDER HARMONICS IN ARTIFICIALLY STACKED CRYSTALS.

The wave propagation of mid-IR pulse and the generated high harmonics is calculated with the reported optical constants for WS_2 in the range from 365 nm to 1700 nm [1]. For the 5μm driver the closed parameters we found in the literature are n = 2.73, k = 0 for a mid-IR

wavelengths of 1.7 μm [2]. The simulated results are displayed in Fig. 2 for various high harmonics. The top panels show the response for $k = 0$, with absorption only, the middle panel for $\Delta n = 0$, i.e., phase matching only. The bottom panel includes both, reduction due to phase matching and absorption effects. The red curve shows the quadratic response for coherent interference enhancement assuming zero phase delay and no reabsorption for HHG from all layers. At few layer numbers, the simulation is close to the quadratic response. With increasing thickness of the AA stacked lattice and for harmonics above the band gap, phase mismatch and reabsorption of HHG light become significant, and the response deviates from quadratic coherent enhancement.

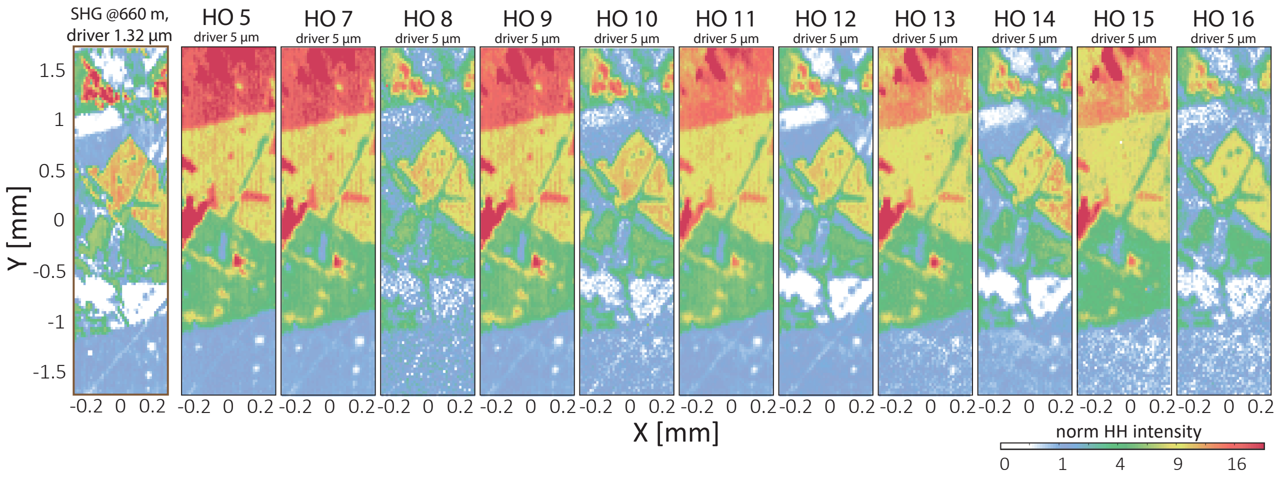

FIG. 1. **SHG and HHG mapping for all measured harmonics.** Using the signal of the OPA centered around 1.32 μm, SHG is measured from the artificial stacked sample. The SHG map shows similar results as the even order harmonics, i.e., HO: 8, 10, 12, 14 and 16. Different domains such as AA and AB stacking can be assigned, see main manuscript. Odd order harmonics are able to probe the number of stacked layers, independent on their AA, AB stacking orientation.

[1] Georgy A. Ermolaev, Dmitry I. Yakubovsky, Yury V. Stebunov, Aleksey V. Arsenin, and Valentyn S. Volkov, "Spectral ellipsometry of monolayer transition metal dichalcogenides: Analysis of excitonic peaks in dispersion," Journal of Vacuum Science and Technology B **38**, 014002 (2020)

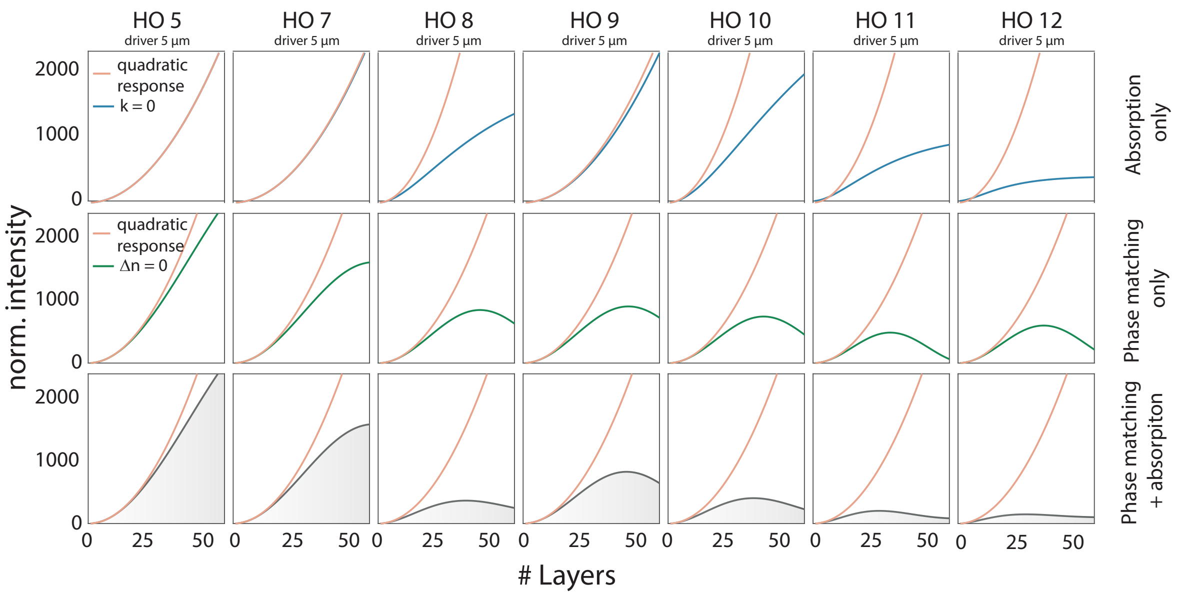

FIG. 2. **Generation and propagation of high-order harmonics.** Simulation of the HHG intensity enhancement of various AA stacked WS_2 stacked layers. The red line is quadratic as a function of the number of layers, assuming no phase delay or reabsorption; the gray line is simulation of the HHG intensity based on real (n) and imaginary refractive (k) indices of TMDC crystal, the blue line takes only n into account, whereas the green line takes only k into account.

.

[2] Hsiang-Lin Liu, Teng Yang, Jyun-Han Chen, Hsiao-Wen Chen, Huaihong Guo, Riichiro Saito, Ming-Yang Li, and Lain-Jong Li, "Temperature-dependent optical constants of monolayer MoS2, MoSe2, Ws2, and WSe2: spectroscopic ellipsometry and first-principles calculations," Scientific Reports **10** (2020).
